# Association of Recorded Cerebrospinal Fluid Diversion with Registry Outcome Status in Pediatric Medulloblastoma: An Exploratory 10-Year Single-Centre Cohort Study

**DOI:** 10.3390/jcm15176544

**Published:** 2026-08-25

**Authors:** Radu-Eugen Rizea, Karina Lidia Gheorghita

**Affiliations:** 1 Department of Neurosurgery, “Carol Davila” University of Medicine and Pharmacy, 050474 Bucharest, Romania; 2 Department of Neurosurgery, Bagdasar-Arseni Clinical Emergency Hospital, 041915 Bucharest, Romania; 3 Private Practice Pediatric Neurology, 031871 Bucharest, Romania; karina.gheorghita@gmail.com

**Keywords:** pediatric medulloblastoma, hydrocephalus, cerebrospinal-fluid diversion, retrospective cohort, registry outcome, metastatic disease, exploratory logistic regression

## Abstract

**Objectives.** To examine, in an exploratory institutional cohort, whether a registry record of cerebrospinal fluid (CSF) diversion was associated with the available binary registry outcome status in children with medulloblastoma, while distinguishing this intervention-related variable from radiographic hydrocephalus. **Methods.** We performed a retrospective single-institution cohort study of 85 patients younger than 18 years with histologically confirmed medulloblastoma who underwent surgical treatment at the Neurosurgery Unit of Bagdasar-Arseni Emergency Hospital. The exposure was any source-record indication of external ventricular drainage, shunting, or ventriculocisternostomy. The analytic file did not provide the timing, indication, or standardized type of diversion; therefore, the exposure could not be characterized as occurring at presentation or before tumor resection. The outcome was the database field labelled “five-year survival,” coded as alive or death/event. Dates of diagnosis, death, progression, and last follow-up were unavailable, so uniform five-year ascertainment and the meaning of “event” could not be verified. Descriptive analyses, univariable comparisons, and a parsimonious logistic model including recorded CSF diversion and metastatic disease were performed. **Results.** The median age was 6 years (IQR, 3–11), and 47 patients were male (55.3%). Hydrocephalus was recorded in 67 patients (78.8%), any CSF diversion in 40 (47.1%), metastatic disease in 12 (14.1%), and gross-total resection in 64 (75.3%). The registry outcome was recorded as alive in 56 patients (65.9%) and death/event in 29 (34.1%). The recorded death/event category occurred in 24/40 patients with diversion versus 5/45 without diversion (60.0% vs. 11.1%; *p* < 0.001), and in 9/12 patients with metastasis versus 20/73 without metastasis (75.0% vs. 27.4%; *p* = 0.002). In the mutually adjusted exploratory model, recorded CSF diversion was associated with the death/event category (OR, 12.71; 95% CI, 3.75–43.05; *p* < 0.001), as was metastatic disease (OR, 8.93; 95% CI, 1.66–47.91; *p* = 0.011). **Conclusions.** Recorded CSF diversion was associated with adverse registry outcome status after mutual adjustment for metastatic disease. Given uncertainty regarding timing and indication, possible outcome misclassification, the small number of recorded events, and absent clinical, treatment, and molecular covariates, this finding should be interpreted as an exploratory institutional association that may reflect disease severity and management selection, not procedural causality or validated prognostic value.

## 1. Introduction

Although Medulloblastoma is the most common malignant brain tumor among children, the complexity of its biological characteristics, clinical behavior and responses to treatments makes this an increasingly challenging disease. Transcriptional profiling and subsequent classification into four major categories of molecular groupings–WNT, SHH, Group 3 and Group 4–indicate heterogeneity within each grouping. In addition, there is evidence of even more levels of heterogeneity such as lncRNAs which are related to subgroup specific immune cell infiltration and prognosis [1]. More recent studies have also indicated that the clinical homogeneity of Group 4 tumors will no longer be assumed due to demonstration of clinical actionable heterogeneity in a previously considered intermediate risk category [2].

Additionally, molecular biology has provided insight into how to interpret classical high-risk features. Children who experience amplifications in their MYC or MYCN genes may present with different clinical trajectories based upon the method of amplification as well as genetic alterations associated with the amplification [3]. On the other hand, children who have inherited mutations in their DNA repair mechanisms, such as those involved in Fanconi’s Anemia, often develop poor outcomes from disease caused by aberrant activation of the SHH pathway, regardless of the current treatment modalities [4]. This divergence in treatment paradigms is due to favorable clinical behaviors of WNT-driven tumors in treatment deintensification protocols and the necessity for intensified or new therapies in more aggressive subgrouped tumors [5].

Radiotherapeutic techniques are evolving. Proton therapy and new dose sculpting methods utilize lower doses of radiation to reduce long term neurocognitive, endocrine and vascular morbidities while attempting to maintain local control as stated in several recent comparative analyses [6]. However, utilization of these advanced radiotherapeutic techniques varies greatly around the world and particularly so in low-and-middle income countries. Disparities in patient outcomes are significant across all countries due to delayed referrals to medical facilities, complete failure to deliver prescribed treatments, lack of availability of radiotherapy services and abandonment of treatments initiated [7]. For this reason, institutional series remain relevant when they report detailed information regarding clinical presentation, surgical interventions and post-treatment follow-up in a transparent manner.

At some hospitals, molecular subgrouping is not immediately available at the bedside, and routinely recorded clinical variables remain useful for describing presentation and patient management. Tumors in the posterior fossa can obstruct cerebrospinal fluid (CSF) pathways and cause ventricular enlargement, increased intracranial pressure, optic disc swelling, vomiting, or worsening consciousness. However, CSF diversion is not simply a baseline biological feature. Whether to insert an external ventricular drain, place a shunt, or perform ventriculocisternostomy depends on neurological status, ventricular size, tumor anatomy, the timing of definitive surgery, surgeon preference, and institutional practice. In this registry, diversion was recorded separately from hydrocephalus, but its timing and indication were not standardized. Thus, recorded diversion is treated here as an intervention-related exposure and as a possible surrogate for disease severity or management selection, rather than as evidence of very severe obstruction at presentation.

This study examines a consecutive cohort of children with medulloblastoma treated over a ten-year period. It focuses on the association between registry variables and the binary registry outcome field. Because the clinical registry lacks time-to-event data and major clinical, treatment, and molecular determinants that influence outcome, this analysis is exploratory and is not intended to develop or validate a clinical prognostic model. The primary aim was to determine whether recorded CSF diversion was associated with the registry outcome category after adjustment for metastatic disease. The secondary aim was to describe the relationship between hydrocephalus identified on imaging and recorded diversion, without assuming that diversion occurred at presentation, preceded tumor resection, or reflected uniform clinical indications.

## 2. Materials and Methods

### 2.1. Study Design, Setting and Ethical Framework

We conducted a retrospective, single-institution cohort study of patients with histologically confirmed medulloblastoma who underwent surgical treatment at the Neurosurgery Unit of Bagdasar-Arseni Emergency Hospital. Patients were identified from the institutional registry and hospital records, and pre-existing clinical, operative, and imaging data were reviewed. The study was conducted in accordance with the applicable institutional requirements for retrospective use of clinical data. No temporal classification was inferred from the name of the procedure or from the presence of hydrocephalus alone. The study was approved by the Ethics Committee of Bagdasar-Arseni Emergency Clinical Hospital, Bucharest (approval no. 56830/02.12.2024, dated 2 December 2024). Given the retrospective design and the use of pre-existing clinical records, the requirement for individual informed consent was waived by the Ethics Committee.

### 2.2. Cohort Assembly and Eligibility Criteria

**Figure 1:**
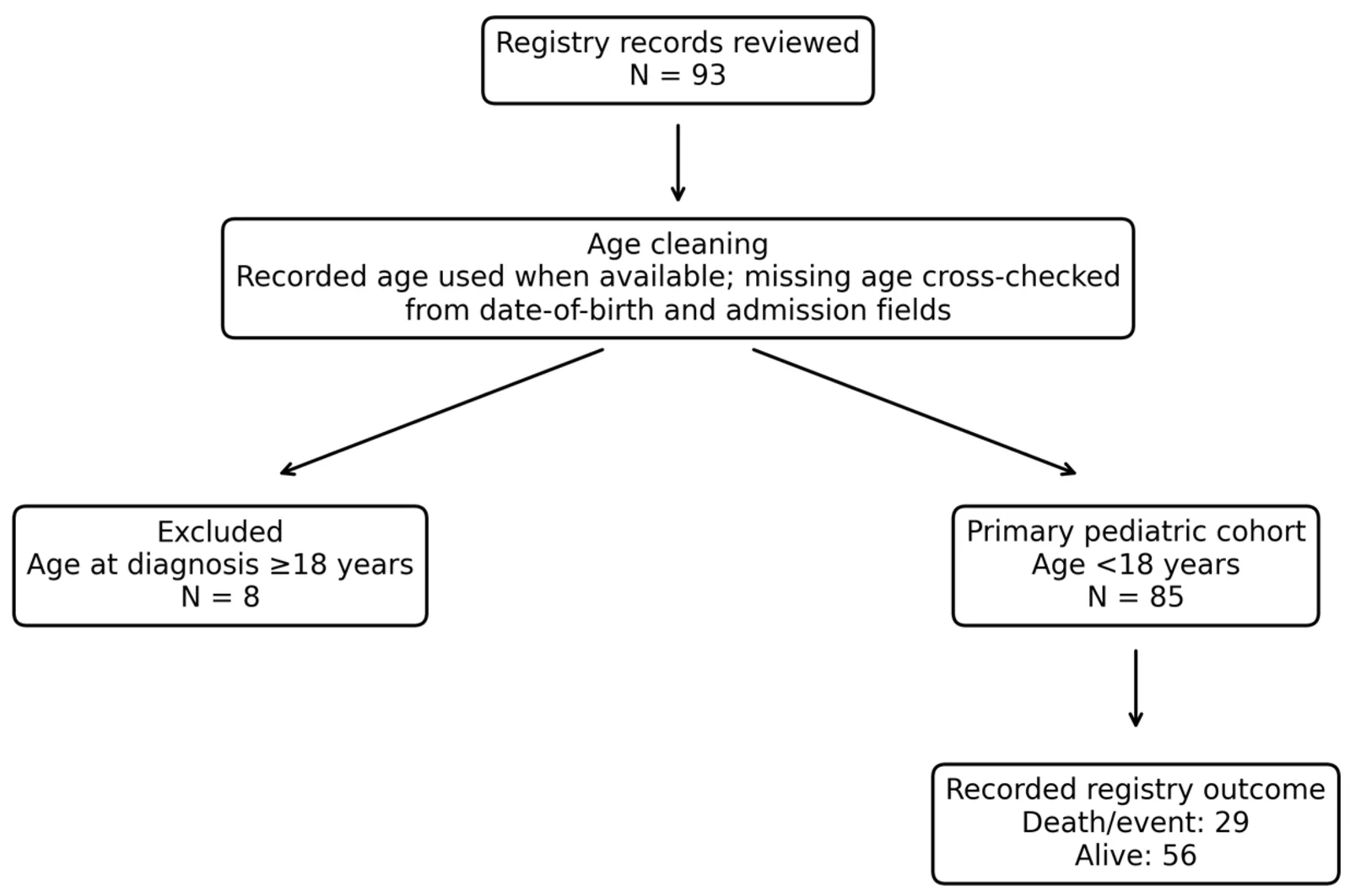
Cohort assembly and analytic flow. The primary analysis was restricted to patients younger than 18 years after age verification and eligibility assessment.

The institutional registry contained 93 medulloblastoma records. Missing ages were cross-checked against available date-of-birth and admission fields. Eight records were excluded because age at diagnosis was 18 years or older after cleaning, and 85 patients were retained in the pediatric analysis. The recorded outcome category was alive in 56 patients and death/event in 29. Figure 1 summarizes the analytic flow.

### 2.3. Variables and Definitions

The available variables were age at diagnosis, sex, hydrocephalus, recorded CSF diversion, metastatic disease at diagnosis, and gross-total resection. Hydrocephalus was a binary registry variable derived from the source record. Recorded CSF diversion was defined broadly as any entry in the diversion field indicating external ventricular drainage, shunting, or ventriculocisternostomy. The analytic file did not provide a standardized procedure date, indication, or sequence relative to tumor resection; consequently, diversion could have occurred before resection, perioperatively, or later for persistent or postoperative hydrocephalus. Diversion type was not sufficiently standardized for reliable type-specific analysis. The variable was therefore analysed as “any recorded CSF diversion,” not as diversion “at presentation” or an urgent preoperative intervention. A stricter sensitivity variable included only explicit “yes” entries in the drainage field. Metastatic disease and gross-total resection were coded from their dedicated registry fields. Gross-total resection was counted only when the dedicated field contained an explicit affirmative response.

Histological subtype was recoded descriptively from heterogeneous free-text entries. Standardized patient-level molecular subgroup, MYC/MYCN amplification, TP53 status, tumor size, fourth-ventricular obstruction, papilledema, consciousness level, preoperative deterioration, residual tumor volume, treatment completion, and radiotherapy timing were not available in a complete linkable format and were not modelled. Surgeon preference and institutional decision pathways were also unavailable. These omissions preclude separation of biological severity from management selection.

Gross-total resection was coded from the dedicated registry field. The analytic file did not specify whether this classification was based on the operative report, postoperative MRI, CT, or another standardized method. It is therefore reported as registry-recorded gross-total resection.

Patient-level data on craniospinal irradiation, radiation dose and timing, chemotherapy regimen, treatment initiation, treatment completion, treatment interruption, and treatment abandonment were not available.

### 2.4. Endpoint Definition

The analytic file contained a binary field labelled “five-year survival,” with categories recorded as alive or death/event. Exact dates of diagnosis, death, progression, and last follow-up were unavailable, as was the method by which the field had been updated. We therefore could not verify that every patient had at least five years of ascertainment, determine how patients lost before five years were handled, confirm that patients recorded alive were assessed at a common landmark, or determine whether “event” denoted death alone or also included progression. Throughout the revised manuscript, the endpoint is therefore termed “recorded registry outcome status,” with the categories “recorded alive” and “recorded death/event.” It must not be interpreted as a formally censored five-year overall-survival or progression-free-survival outcome.

### 2.5. Statistical Analysis

Statistical analyses were performed using Python, version 3.13.5 (Python Software Foundation, Wilmington, DE, USA), with the pandas, NumPy, SciPy, and statsmodels packages.

Age was summarized using the median and interquartile range (IQR), while categorical variables were reported as counts and percentages. For comparisons of categorical variables with the recorded death/event category, the chi-square test or Fisher’s exact test was applied when expected cell counts were less than five. Risk ratios (RRs) and odds ratios (ORs), with 95% confidence intervals (CIs), were calculated as estimates of association. To evaluate differences in age between the recorded outcome groups, a two-tailed Mann–Whitney U test was used. These statistical tests provide information only regarding associations within the registry-collected data and do not permit conclusions regarding causation.

Cramer’s V was used to assess relationships among binary variables. Because only 29 cases were recorded in the death/event category, and because hydrocephalus and diversion represent related constructs, the primary multivariable adjustment included only two variables: metastatic disease and any recorded CSF diversion. Logistic regression was employed to estimate mutually adjusted ORs for the recorded death/event category. A separate sensitivity analysis replaced the broad definition of any recorded CSF diversion with a stricter variable coded only when the source field explicitly indicated drainage. Given the limited number of adverse outcomes, together with uncertainty regarding exposure timing and outcome ascertainment, no clinical prediction score, calibration analysis, decision-curve analysis, or claims of predictive performance were presented. Resampling methods were not used because they cannot correct for exposure misclassification, outcome uncertainty, or omitted-variable bias.

Logistic regression was selected because the only available outcome was a dichotomous registry field. Dates of diagnosis, death, progression, and last follow-up were unavailable; therefore, Kaplan–Meier or Cox proportional-hazards analyses could not be performed. The logistic model estimates the odds of belonging to the recorded death/event category and does not estimate a five-year survival probability or a time-to-event effect.

## 3. Results

### 3.1. Cohort Flow and Eligibility

The institutional registry contained 93 medulloblastoma records. After cleaning the age variable and applying the pediatric eligibility criterion, 85 patients were retained in the primary analysis. The remaining records were adult/young-adult cases or records with unresolved age data and were not included in the main pediatric cohort. Figure 1 summarizes the analytic flow. The final cohort size is consistent across all tables, figures and multivariable analyses.

### 3.2. Baseline Characteristics

**Figure 2:**
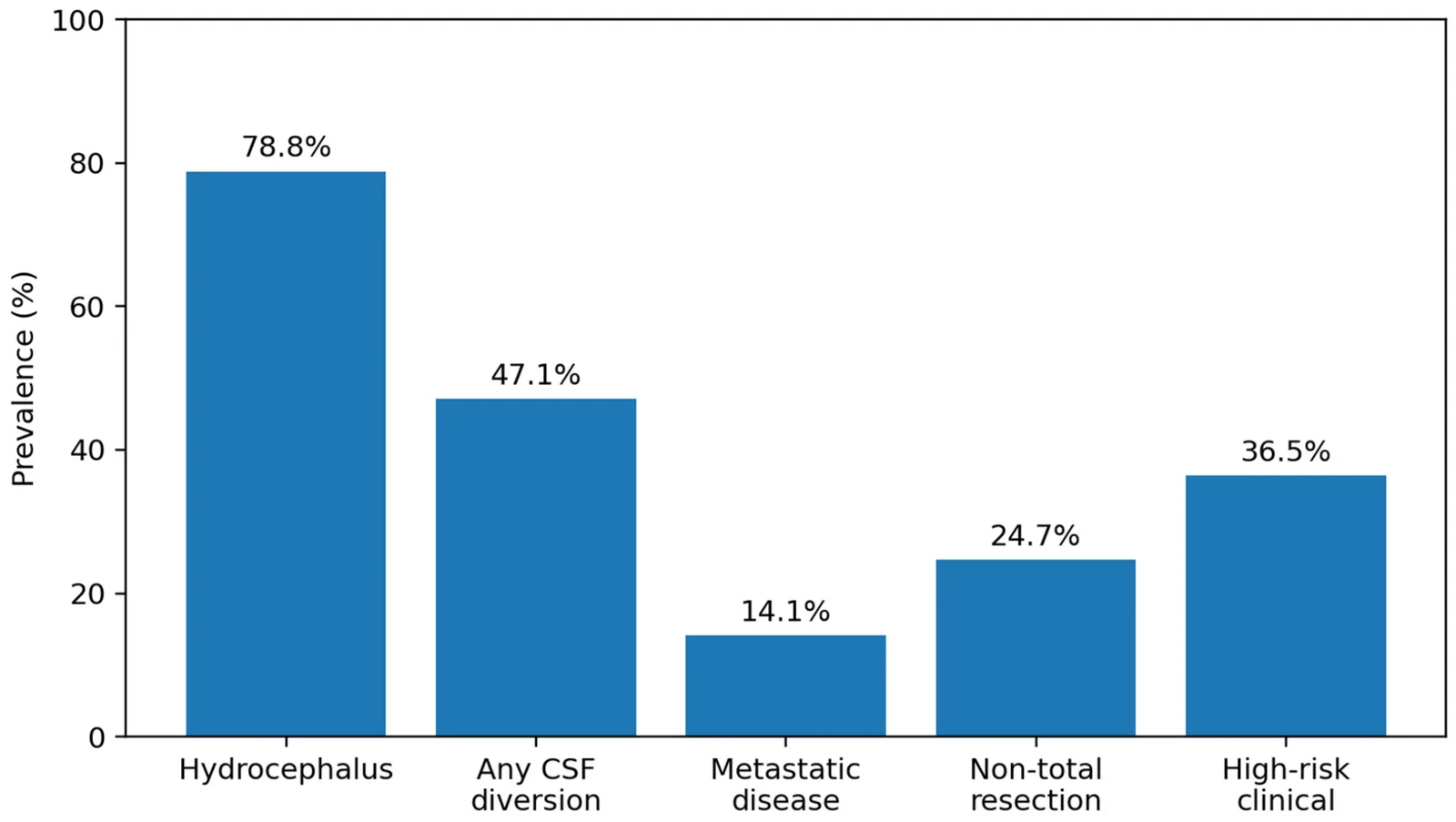
Prevalence of selected clinical and treatment-related characteristics in the study cohort (N = 85). Hydrocephalus refers to radiographically documented hydrocephalus. Any CSF diversion denotes any source-record indication of external ventricular drainage, shunt placement, or ventriculocisternostomy. Non-total resection includes all procedures not recorded as gross-total resection. The final category represents an exploratory composite defined as metastatic disease and/or non-total resection and should not be interpreted as a validated clinical risk classification.

The median age at diagnosis was 6 years (IQR, 3–11; range, 1–17). Male patients represented 47/85 (55.3%) of the cohort. Hydrocephalus was recorded in 67/85 (78.8%) patients, and any CSF diversion was recorded in 40/85 (47.1%). Metastatic disease at diagnosis was present in 12/85 (14.1%), and gross-total resection was recorded in 64/85 (75.3%). The registry outcome category was recorded alive in 56/85 (65.9%) and death/event in 29/85 (34.1%) (Table 1). Baseline distributions are shown in Figure 2.

### 3.3. Univariable Associations with Recorded Registry Outcome

**Figure 3:**
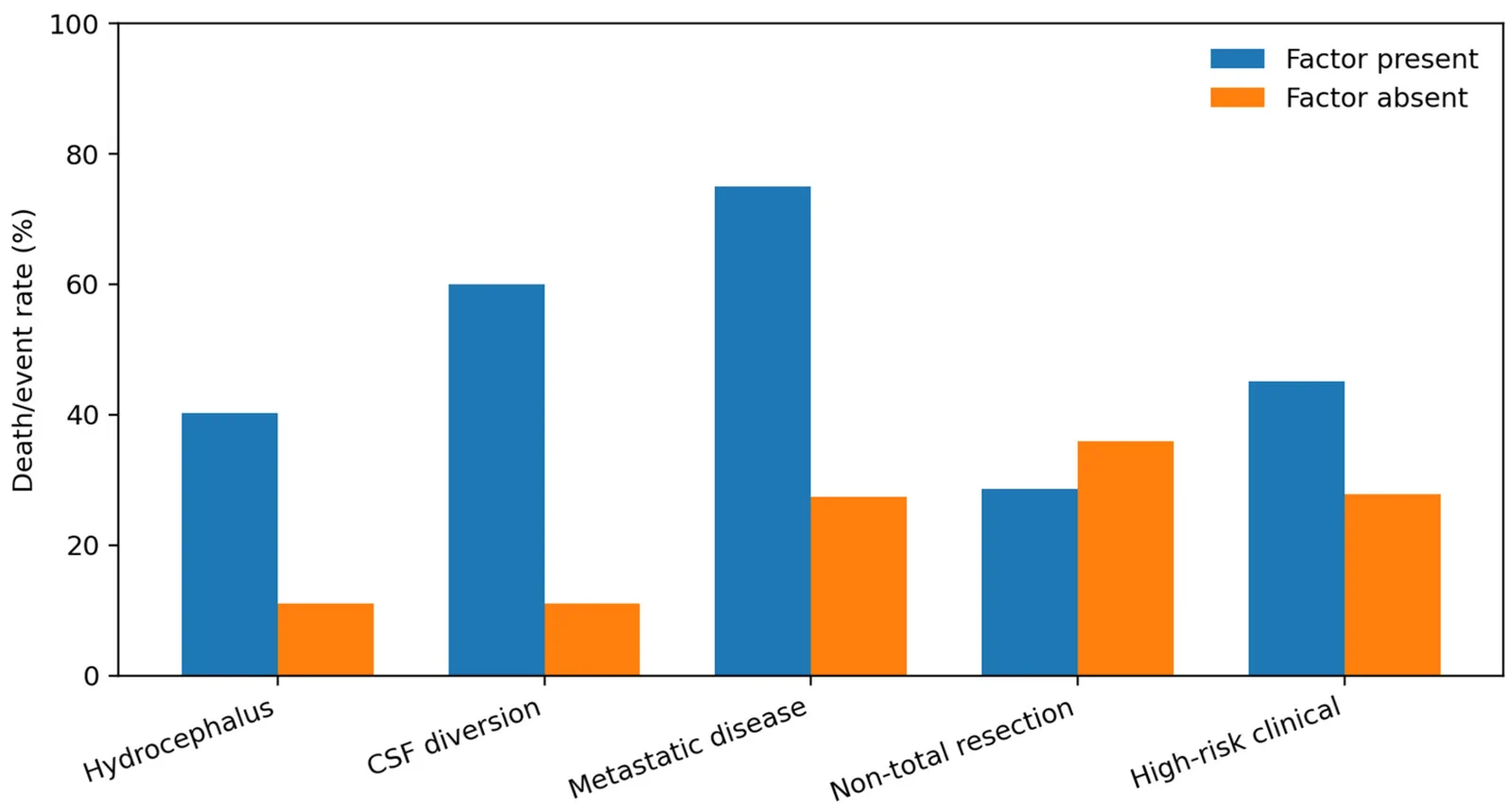
Recorded death/event proportions according to available clinical factors. The figure displays crude registry associations and should not be interpreted causally.

Patients with any recorded CSF diversion had a higher proportion in the recorded death/event category than those without diversion: 24/40 (60.0%) versus 5/45 (11.1%; *p* < 0.001). Metastatic disease was also associated with the recorded outcome: 9/12 (75.0%) patients with metastasis were in the death/event category compared with 20/73 (27.4%) without metastasis (*p* = 0.002). Hydrocephalus showed a weaker univariable association, while sex and gross-total resection were not statistically significant in this dataset. Table 2 summarizes these comparisons, and Figure 3 displays the crude outcome proportions.

Age was not significantly different between registry outcome categories. Median age was 5 years (IQR, 3–7) among patients recorded as death/event and 7 years (IQR, 4–13) among those recorded alive (Mann–Whitney *p* = 0.057).

### 3.4. Inter-Variable Relationships

**Figure 4:**
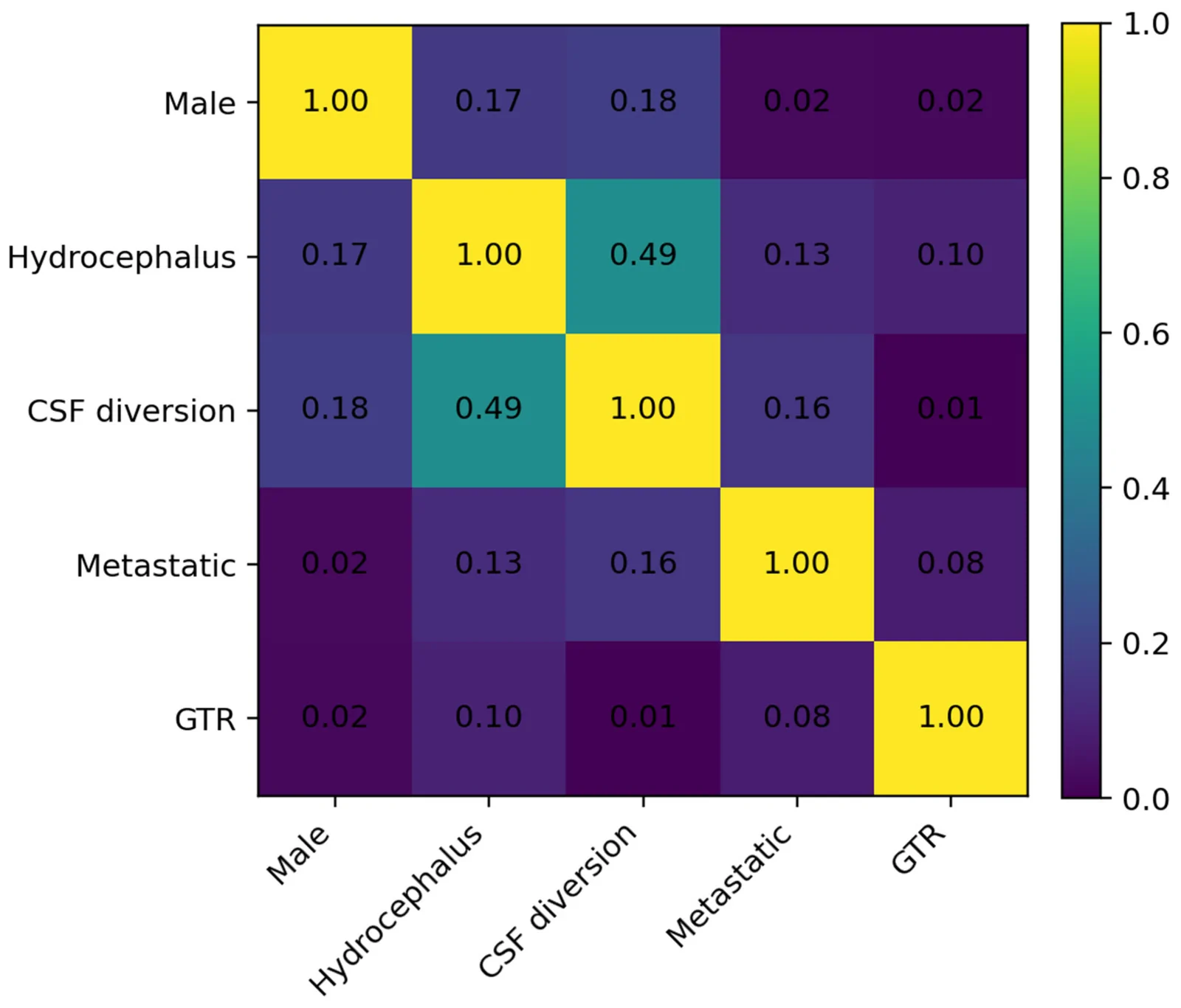
Cramer’s V heatmap for binary predictors. Values close to 0 indicate weak association and values close to 1 indicate stronger association. The diagonal is 1 by definition.

Cramer’s V analysis showed a moderate relationship between hydrocephalus and recorded CSF diversion (V = 0.49), confirming that the variables overlap but are not identical (Figure 4). This relationship does not establish that diversion represented a uniform severity threshold, because timing, indication, neurological status, ventricular measurements, and management preferences were unavailable. To limit construct overlap and overfitting, hydrocephalus was not included in the parsimonious primary adjusted model.

### 3.5. Exploratory Adjusted Association Analysis

The parsimonious exploratory model included any recorded CSF diversion and metastatic disease. After mutual adjustment, recorded CSF diversion remained associated with the recorded death/event category (OR, 12.71; 95% CI, 3.75–43.05; *p* < 0.001), and metastatic disease was also associated with the outcome category (OR, 8.93; 95% CI, 1.66–47.91; *p* = 0.011). These estimates are presented in Table 3. The wide confidence intervals and unmeasured confounding preclude interpretation as validated prognostic effects.

### 3.6. Sensitivity Analysis and Scope of Inference

Using the strict drainage definition, 24/38 (63.2%) patients with an explicit affirmative drainage entry were in the recorded death/event category compared with 5/47 (10.6%) without such an entry (univariable OR, 14.40; 95% CI, 4.62–44.92; *p* < 0.001). In a two-variable model with metastatic disease, strict drainage remained associated with the recorded death/event category (OR, 14.95; 95% CI, 4.38–51.05; *p* < 0.001), while metastatic disease had an OR of 8.63 (95% CI, 1.56–47.78; *p* = 0.014).

A broader model containing age, sex, hydrocephalus, recorded diversion, and metastatic disease produced directionally similar estimates but very wide confidence intervals. Because the cohort contained only 29 records in the death/event category and lacked standardized exposure timing, endpoint ascertainment, molecular subgroup, residual tumor volume, and treatment variables, the broader model is not emphasized. Accordingly, no prediction score or assessment of clinical utility is presented, and the adjusted estimates should be regarded solely as exploratory measures of association.

## 4. Discussion

There were 56/85 (65.9%) patients in the single-center pediatric medulloblastoma cohort who were classified into the “alive” category of the registry outcome measure, and there were 29/85 (34.1%) who were classified into the “death/event” category of the registry outcome measure. The association between recorded CSF diversion and the adverse registry category remained after adjustment for metastatic disease. There was a weaker crude association between hydrocephalus and the adverse registry category.

These results report an association within an institutional registry and do not indicate that diversion occurred at the onset of the disease, that diversion can independently predict survival, or that diversion can alter the course of the disease.

It is critical to interpret the association between cerebrospinal fluid (CSF) diversion and the registry outcome with caution. The decision to divert CSF is typically made as part of the treatment plan and may serve as a proxy for several other variables, such as the size of the ventricles, the degree of neurological impairment, elevated intracranial pressure due to tumor compression, tumor shape and size, the immediacy of definitive surgery, surgeon preference, and local practices. This presents a high risk of confounding by indication because patients selected to undergo diversion may vary systematically from those treated by initial surgery or observational management in terms of their neurological function, ventricular size, anatomical blockage, timing of referral, and perioperative risk. The registry did not differentiate among types of diversion performed before surgery, during or immediately following surgery, or subsequent to surgery as treatment for chronic postsurgical hydrocephalus. Therefore, each scenario represents a different state of disease or complication. Thus, the observed odds ratio may reflect unmeasured clinical severity and/or selection of management rather than an independent prognostic contribution of the diversion variable.

In the classical model, posterior fossa tumors produce obstructive hydrocephalus by narrowing the fourth ventricular outlets or other CSF pathways and impairing bulk CSF circulation. Contemporary concepts view CSF homeostasis as a distributed process involving exchange across capillary, interstitial, ependymal, glymphatic, and lymphatic pathways, together with venous pressure, intracranial compliance, and pulsatility [8]. Ventricular enlargement in children with posterior fossa tumors may therefore reflect more than a single fixed point of obstruction. These mechanisms were not measured in the present database and cannot be inferred from the recorded diversion variable.

Metastatic disease is known to be an unfavorable determinant in pediatric medulloblastoma and continues to play a central role in clinical risk stratification [9,10,11]. After mutual adjustment for recorded diversion in the current dataset, metastasis was associated with the death/event category. This consistency indicates that metastasis should continue to be retained as an essential covariate; however, it does not address concerns regarding the registry endpoint and the lack of molecular and treatment data.

Comparing the proportion recorded as alive in this registry with five-year survival rates from cooperative trials or population-based datasets is inappropriate. The analytic file did not contain dates of diagnosis, death, progression, or last follow-up; thus, uniform five-year ascertainment could not be confirmed. Additional differences in molecular subtypes, radiotherapy availability and timing, chemotherapy completion, treatment interruptions, referral patterns, and follow-up restrict cross-dataset comparisons [7,9,12,13].

The inclusion period spanned ten years and likely included changes in surgical techniques, radiotherapy techniques, chemotherapy agents, supportive care, and molecular classifications. Due to missing standardized dates of diagnosis and treatment, calendar period and treatment era could not be incorporated into the analysis. Therefore, it is possible that changes in clinical care over the inclusion period contributed, to some extent, to the observed association.

Several unmeasured factors may contribute to the association described above, including tumor size, fourth ventricular obstruction, peritumoral edema, papilledema, level of consciousness, preoperative deterioration, postoperative complications, residual tumor volume, and delayed or incomplete adjuvant therapy. Molecular subgroup, MYC/MYCN amplification, TP53 status, and standardized imaging parameters were also not captured. Since all of these variables were absent from the dataset analyzed above, it is impossible to determine whether the observed association reflects anatomical obstruction, tumor biology, treatment selection, or postoperative morbidity.

The literature describing hydrocephalus management in pediatric medulloblastoma has largely examined factors related to shunting [14], with limited adult evidence linking shunt placement or performance status with outcome [15]. The present study adds one more small institutional registry observation using hydrocephalus and recorded diversion as distinct fields. However, since both the timing and indications for diversion were unknown, we cannot define an anatomical obstruction threshold for clinical use or assign prognostic value to diversion.

Additional studies published recently add context. A molecularly annotated medulloblastoma cohort demonstrated differences in CSF diversion rates based upon molecular subtype; specifically, less diversion was required in WNT tumors [16], which suggests a possible link between diversion requirements and tumor biology. A 2023 medulloblastoma-specific study examining EVD placement and management emphasized that both procedural practices and drain management may affect complications and postoperative hydrocephalus [17]. Additionally, another recent cohort study examining medulloblastoma identified factors associated with the necessity of permanent postoperative diversion, including tumor volume and CSF channel invasion, and supported the conclusion that routine prophylactic permanent diversion is not indicated [18]. A large international multicenter study of pediatric posterior fossa tumors identified no statistically significant difference according to the choice of preoperative hydrocephalus management—EVD, ETV, or tumor resection alone—with regard to persistent postoperative hydrocephalus requiring surgical intervention [19]. While these studies primarily investigated hydrocephalus management rather than long-term oncologic survival, they emphasize how tumor anatomy, biology, patient selection, local protocols, and confounding by indication may affect the interpretation of associations derived from diversion.

The diversion exposure also combines clinically distinct procedures. External ventricular drainage is generally temporary and is commonly used for acute intracranial-pressure control, whereas ventriculoperitoneal shunting usually represents permanent diversion. Pooling these procedures therefore introduces substantial exposure heterogeneity. Because procedure type, timing, and indication were not recorded consistently, modality-specific associations could not be estimated reliably.

Molecular risk stratification is now crucial. WNT medulloblastoma often has a better prognosis; Group 3 disease is commonly metastatic and adversely affects prognosis; Group 4 is heterogeneous; and SHH prognosis depends heavily upon TP53 and MYCN status [1,5,10,11]. Based upon the current dataset, recorded CSF diversion cannot be interpreted apart from current molecular risk stratification. It might best be viewed as identifying a supplementary clinical-management phenotype whose incremental prognostic value should be prospectively assessed in cohorts with complete molecular, imaging, staging, and treatment data.

The revised statistical strategy utilizes exploratory association estimation. A simple two-predictor logistic regression model was utilized because only 29 records were within the adverse outcome category. Wide confidence intervals reflected considerable uncertainty in the adjusted estimates. Bootstrapped internal validation, cross-validation, calibration analysis, a clinical score, and decision-curve analysis were eliminated since none of these strategies can correct for omitted-variable bias, uncertainty in outcome ascertainment, or misclassification of diversion timing and indication. The reported odds ratios represent descriptive adjusted associations; they are not a validated predictive model. Results should therefore be considered exploratory and hypothesis-generating and should not be applied directly to clinical decision-making.

This study has major limitations. First, it is retrospective. Second, it was conducted at a single center. Third, only 29 records were in the death/event category. Fourth, recorded CSF diversion lacked standardization regarding timing, indication, and procedure type and may have included preoperative, perioperative, or postoperative procedures. Fifth, diversion decisions were based on multiple factors, including neurological status, ventricular size, tumor anatomy, surgeon preference, institutional practice, referral timing, and the timing of definitive surgery. Sixth, none of the previously listed variables were available for adjustment. Seventh, residual confounding, likely including confounding by indication, is substantial. Eighth, the registry field labeled “survival at five years” lacked dates of diagnosis, death, progression, and last follow-up; uniform five-year ascertainment, the handling of loss to follow-up, and the distinction between death and progression could not be verified. Ninth, the ten-year period encompassed changes in surgery, radiotherapy, chemotherapy, supportive care, and molecular classification. Tenth, molecular subgroup, MYC/MYCN and TP53 status, residual tumor volume, tumor size, fourth ventricular obstruction, treatment completion, radiotherapy timing, and standardized staging were unavailable. Eleventh, residual confounding and exposure–outcome misclassification are substantial.

Adjuvant treatment is a major determinant of medulloblastoma outcome. The absence of patient-level information on radiotherapy and chemotherapy prevents adjustment for treatment heterogeneity and represents a major source of residual confounding. The observed registry associations may therefore partly reflect differences in treatment access, timing, completion, or interruption.

Therefore, future prospective data collection should include the date and reason for every CSF diversion performed; the relationship between diversion and resection; standardized ventricular and tumor measurements; neurological status; papilledema; level of consciousness; preoperative deterioration; postoperative complications; date of diagnosis; date of surgery; date of last follow-up; date and cause of death; date of progression; M stage; postoperative residual tumor volume; molecular subgroup; MYC/MYCN amplification; TP53 status; completion of treatment; radiotherapy timing; treatment-era variables; institutional protocols; and the clinical criteria used for selecting EVD, ETV, shunting, resection, or observation.

Prospective longitudinal follow-up will remain important. Posterior fossa medulloblastoma survivors experience continued risks of late mortality, seizures, second cancers, vascular events, and neuropsychological sequelae [20]. Therefore, longitudinal follow-up with time-to-event analysis cannot be inferred from the binary registry field employed herein.

## 5. Conclusions

Any recorded CSF diversion and metastatic disease were associated with the recorded death/event category in this exploratory pediatric medulloblastoma cohort. Because the timing and indication of diversion, uniform outcome ascertainment, and major clinical, molecular, and treatment covariates were unavailable, recorded diversion should not be characterized as occurring at presentation, interpreted as a causal exposure, or presented as a validated prognostic marker. The finding is best regarded as a hypothesis-generating institutional association requiring prospective, time-resolved, and molecularly annotated validation.

## Figures and Tables

**Table 1:** Baseline demographic, clinical, surgical, and registry outcome characteristics of the pediatric cohort. Confidence intervals for proportions are Wilson intervals. The outcome categories reflect the available registry field and are not a formally censored time-to-event endpoint.

Characteristic	n/N or Summary	95% CI/Notes
Age at diagnosis, years	6 (3–11); range 1–17	Median (IQR); range
Age group: <3 years	18 (21.2%)	13.8–31.0%
Age group: 3–14 years	57 (67.1%)	56.5–76.1%
Age group: 15–17 years	10 (11.8%)	6.5–20.3%
Male sex	47 (55.3%)	44.7–65.4%
Hydrocephalus	67 (78.8%)	69.0–86.2%
Any recorded CSF diversion	40 (47.1%)	36.8–57.6%; timing unavailable
Metastatic disease at diagnosis	12 (14.1%)	8.3–23.1%
Gross-total resection	64 (75.3%)	65.2–83.2%
Clinically high-risk: metastatic or non-GTR	31 (36.5%)	27.0–47.1%
Recorded alive category	56 (65.9%)	55.3–75.1%
Recorded death/event category	29 (34.1%)	24.9–44.7%

**Table 2:** Univariable associations with the recorded death/event category. RR and OR are oriented toward the death/event category. The strict drainage variable includes only explicit affirmative entries and is presented as a sensitivity comparison to the broader recorded-diversion definition.

Factor	Recorded Death/Event if Present	Recorded Death/Event if Absent	RR (95% CI)	OR (95% CI)	*p*	Test
Male sex	19/47 (40.4%)	10/38 (26.3%)	1.54 (0.81–2.90)	1.90 (0.75–4.80)	0.173	Chi-square
Hydrocephalus	27/67 (40.3%)	2/18 (11.1%)	3.63 (0.95–13.83)	5.40 (1.15–25.42)	0.020	Chi-square
Any recorded CSF diversion	24/40 (60.0%)	5/45 (11.1%)	5.40 (2.28–12.82)	12.00 (3.90–36.94)	<0.001	Chi-square
Strict drainage field = yes	24/38 (63.2%)	5/47 (10.6%)	5.94 (2.50–14.08)	14.40 (4.62–44.92)	<0.001	Chi-square
Metastatic disease at diagnosis	9/12 (75.0%)	20/73 (27.4%)	2.74 (1.67–4.50)	7.95 (1.95–32.38)	0.002	Fisher exact
Non-total resection	6/21 (28.6%)	23/64 (35.9%)	0.80 (0.38–1.69)	0.71 (0.24–2.09)	0.537	Chi-square
Clinically high-risk: metastatic or non-GTR	14/31 (45.2%)	15/54 (27.8%)	1.63 (0.91–2.90)	2.14 (0.85–5.40)	0.104	Chi-square

**Table 3:** Parsimonious exploratory logistic regression model. Odds ratios are mutually adjusted and oriented toward the recorded death/event category; they do not establish causality or validated prognostic performance.

Predictor	Mutually Adjusted OR for Recorded Death/Event (95% CI)	*p*
Any recorded CSF diversion	12.71 (3.75–43.05)	<0.001
Metastatic disease	8.93 (1.66–47.91)	0.011

## Data Availability

The data presented in this study are available from the corresponding author upon reasonable request, subject to applicable institutional and ethical restrictions. The data are not publicly available due to patient confidentiality and privacy considerations.
